# An integrated clinical and genomic information system for cancer precision medicine

**DOI:** 10.1186/s12920-018-0347-9

**Published:** 2018-04-20

**Authors:** Yeongjun Jang, Taekjin Choi, Jongho Kim, Jisub Park, Jihae Seo, Sangok Kim, Yeajee Kwon, Seungjae Lee, Sanghyuk Lee

**Affiliations:** 1 Ewha Research Center for Systems Biology (ERCSB), Ewha Womans University, Seoul, Korea; 2 Interdisciplinary Program in Bioinformatics, College of Natural Science, Seoul National University, Seoul, Korea; 3 Daumsoft, Inc., Seoul, Korea; 4 DNA Link, Inc., Seoul, Korea

**Keywords:** Information system, Genomic medicine, Personalized medicine, Precision medicine, Targeted therapy, Oncology, Cancer

## Abstract

**Background:**

Increasing affordability of next-generation sequencing (NGS) has created an opportunity for realizing genomically-informed personalized cancer therapy as a path to precision oncology. However, the complex nature of genomic information presents a huge challenge for clinicians in interpreting the patient’s genomic alterations and selecting the optimum approved or investigational therapy. An elaborate and practical information system is urgently needed to support clinical decision as well as to test clinical hypotheses quickly.

**Results:**

Here, we present an integrated clinical and genomic information system (CGIS) based on NGS data analyses. Major components include modules for handling clinical data, NGS data processing, variant annotation and prioritization, drug-target-pathway analysis, and population cohort explorer. We built a comprehensive knowledgebase of genes, variants, drugs by collecting annotated information from public and in-house resources. Structured reports for molecular pathology are generated using standardized terminology in order to help clinicians interpret genomic variants and utilize them for targeted cancer therapy. We also implemented many features useful for testing hypotheses to develop prognostic markers from mutation and gene expression data.

**Conclusions:**

Our CGIS software is an attempt to provide useful information for both clinicians and scientists who want to explore genomic information for precision oncology.

**Electronic supplementary material:**

The online version of this article (10.1186/s12920-018-0347-9) contains supplementary material, which is available to authorized users.

## Background

Deep sequencing is about to become a part of clinical tests, but the probabilistic and complex nature of the results makes it vastly different from conventional clinical tests that are deterministic and simple to use without sophisticated informatics analysis. Systematic interpretation of genomic alterations obtained from NGS data remains challenging especially intended for clinical application. In particular, determining clinical and biological significance of each variant in terms of the diagnostic, therapeutic, and prognostic implications for individual patients poses considerable difficulties due to the inconsistency in biological annotations on human genome, variations, and therapeutics from various parties [1]. Furthermore, the complexity in NGS data analysis procedure makes it unrealistic for practicing oncologists to grasp meanings and uncertainties of the results easily without ongoing education in genomics and bioinformatics. Thus, a systematic and easy-to-understand interpretation system with a readily accessible knowledgebase is urgently needed to identify specific genomic alterations and genotype-matched therapeutic options with clinical relevance, the most critical step in implementing precision oncology.

Recently several groups reported implementation of CGISs which addressed computational and clinical issues involved. PathOS is a web-based CGIS incorporating variant filtering, curation and reporting, but it was mostly for targeted (amplicon) gene sequencing and did not include variant-level recommendation of targeted drugs [2]. CVE was developed as an R package to identify drivers, resistance mechanisms and to assess druggability, but lacks support for patient cohort population [3]. Most systems are focused on either NGS data processing and annotation, or information management issues relevant to clinical applications. Thus, it would be desirable to develop a comprehensive information system that supports diverse features helpful for cancer precision medicine not only for clinical service providers but also for medical scientists. Here, we describe a CGIS implementation of such features and discuss the key bioinformatic challenges in software development.

## Implementation

### Overview of system and features

The aims of our CGIS software are (1) to provide a clinical report of recommended therapies with full variant-level annotation based on NGS data analysis and (2) to support medical scientists for exploring patient cohort data to test hypotheses for developing patient stratification schemes, molecular biomarkers, and alternative treatment options.

**Fig. 1:**
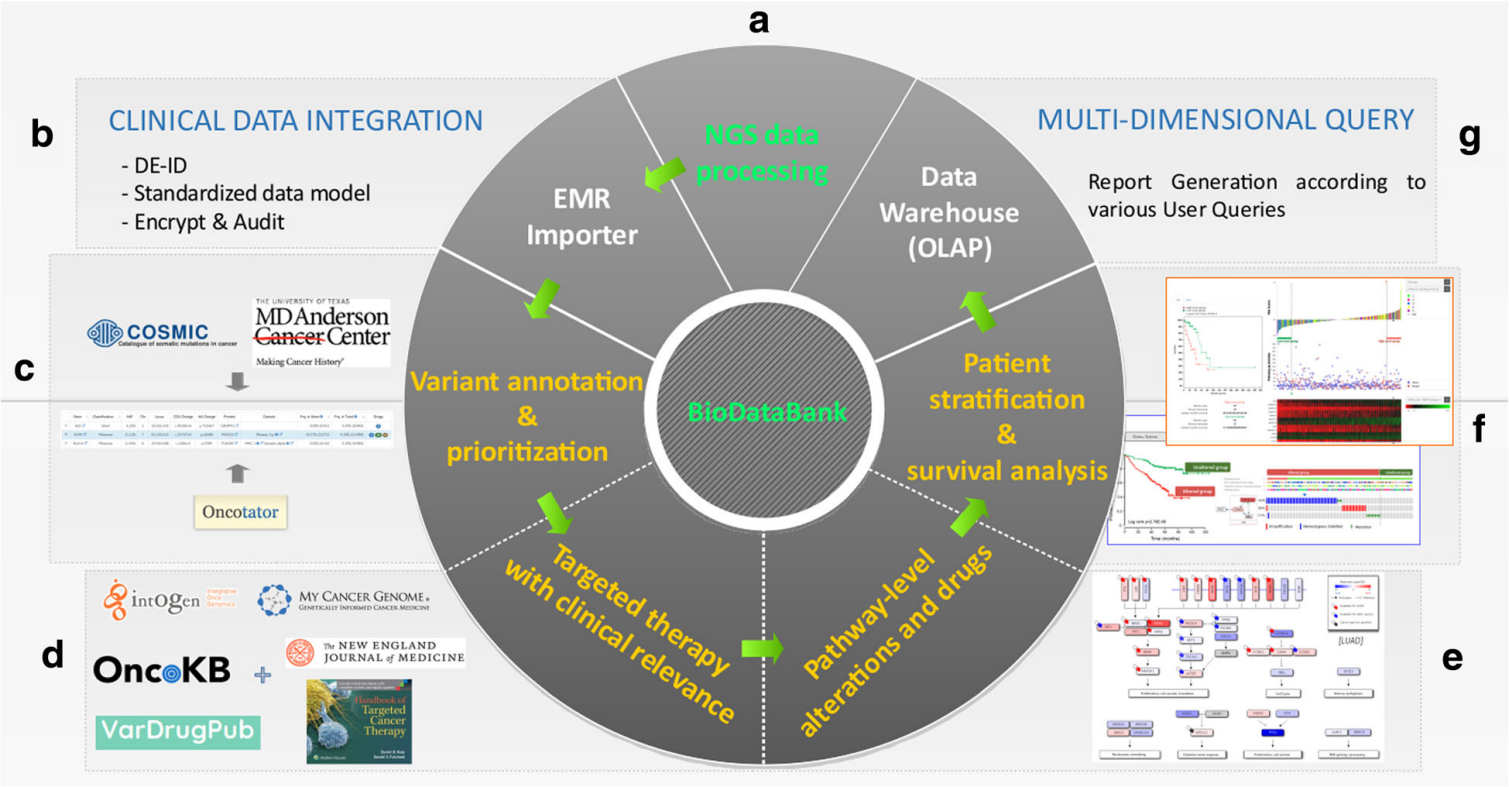
System architecture of our CGIS software. Information flow among various components is indicated in arrows. BioDataBank is the central knowledgebase of information for genes, variants, drugs, curated records, cohort population data, etc. Labels beside component panels (**a**, **b**, **c**, **d**, **e**, **f** and **g**) correspond to description markers in the section of Implementation/Overview of system and features

Representative features are as follows in the order of information processing as summarized in Fig. 1:

A) NGS data processing which includes variant calling from whole exome sequencing (WES) data and expression quantification from whole transcriptome sequencing (WTS, a.k.a. RNA-seq) data. We calculate the somatic single nucleotide variants (SNVs), insertions and deletions (INDELs), and copy number variations (CNVs) using Mutect [4], Strelka [5], and EXCAVATOR [6], respectively. The MapSplice-RSEM [7, 8] pipeline was used for RNA-seq quantification to warrant accuracy in spite of long computation time. Galaxy [9] pipelines for WES and WTS data processing are shown in Additional file 1: Figure S1 and Additional file 2: Figure S2 respectively. We also provide Galaxy workflow files for WES and WTS data processing in Additional files 3 and 4 respectively so that those files can be imported into another Galaxy server. Additionally, users can upload their own FASTQ files into our BioCloud system for processing NGS data and for getting the various reports described below. Step by step demonstration for this procedure is fully described in Additional file 5.
B) Import of clinical information from patient’s medical record, which includes de-identification and encryption using standard data model of NCI Clinical Data Elements (https://gdc.cancer.gov/clinical-data-elements).
C) Variant annotation and prioritization to identify driver alterations or targeted drugs. Genomic alterations were curated at both the gene and variant levels to identify function-affecting variants in cancer genes of the COSMIC database [10].
D) Targeted therapy with clinical relevance to obtain “actionable” targets of different significance. Many curated resources were amassed to establish the list of actionable target genes and variants (i.e. cases where the targeted drugs are available clinically).
E) Pathway view of genomic alterations and available targets. Key pathway genes are manually curated for several cancer types to enhance mechanistic understanding that might lead to alternative therapies.
F) Patient stratification and survival analysis which facilitate medical scientists to test clinical hypotheses for the purpose of developing diagnostic or prognostic molecular markers. We support patient classification by the mutual exclusivity of somatic mutations and by the gene expression signatures.
G) Clinical report system to help clinical decision in an easy-to-use GUI format.

### BioDataBank

High-quality interpretations of individual genomic variants inevitably requires vast amount of information collection, proper data modeling, curation of raw data, and integration to build a comprehensive knowledgebase. BioDataBank is our knowledgebase encompassing gene, protein, gene variants in cancer, population (cohort) data, and drugs for clinical therapy. Table 1 is the list of resources that we integrated to build the BioDataBank. Specifically, cancer gene variants were catalogued from the COSMIC [10] and TCGA databases. Curated information on targeted drugs in clinical use or in clinical trials were amassed from various databases such as OncoKB [11], MyCancerGenome [12], and the Personalized Cancer Medicine Knowledge Base [13] (see Table 1).

**Table 1:** Public omics data and clinical resources

Category	Resource	Comments
Gene	Hugo symbol (http://www.genenames.org)	Gene symbol mapping
	Entrez genes (https://www.ncbi.nlm.nih.gov/gene)	Gene model
Protein	Uniprot (http://www.uniprot.org)	Protein model
	Pfam (http://pfam.xfam.org)	Protein domains
Cancer gene variants	COSMIC cancer gene census [10]	Catalogue of Somatic Mutations in Cancer
	Personalized Cancer Medicine Knowledge Base [13]	MD Anderson Cancer Center
Cancer omics data	The Cancer Genome Atlas (TCGA; http://cancergenome.nih.gov)	Somatic mutations RNA expressions Copy Number Variations Clinical information
Drug and clinical trials	MyCancerGenome [12]	Vanderbilt-Ingram Cancer Center
	OncoKB [11]	Memorial Sloan Kettering Cancer Center
	VarDrugPub [17]	In-house database for mutation-gene-drug relations mined from all the PubMed articles
	IntOGen [15]	Anticancer drugs database
	Handbook of targeted cancer therapy [16]	More than 120 targeted therapy agents for which clinical trial data are available
	The New England Journal of Medicine	Manual search · Key words: breast | lung | glio & cancer & survival & genetic profile · Searching option: past 10 years

### Cohort database and selection of background patients

Patient grouping and management is an essential part of CGIS to identify other patients with similar mutations or gene expression pattern, which can be used to predict the progress of the disease as well as to identify appropriate therapies. For example, identifying patients with similar molecular characteristics makes it possible to interrogate clinical questions like ‘how did the cancer progress?’ and ‘what would be the effective or non-effective treatments?’. Cancer omics data at population scale is also important for patient stratification to identify subtypes on molecular basis. Our cohort database contains the TCGA multi-omics data with clinical information for each patient. It includes SNVs, CNVs, RNA expression data on 1845 tumor and 1929 normal samples across three focused cancer types (breast invasive carcinoma, glioblastoma multiforme, and lung adenocarcinoma) currently. To support researchers to find patient cohorts that meet their study goals, we implemented a filtering scheme to select the patient cohort based on their clinical or molecular features, including histological subtypes, risk factors, mutational features, diagnosis, therapeutic actions, and treatment outcome at an individual patient level. For example, EGFR was the most frequently mutated gene among female and lifelong never-smoker patients, whereas TP53 mutation was prevalent in other patients, which can be readily confirmed using our cohort explorer for the TGCA LUAD cohort (shown in Additional file 6: Figure S4).

## Results

### Variant annotation and Druggability

**Fig. 2:**
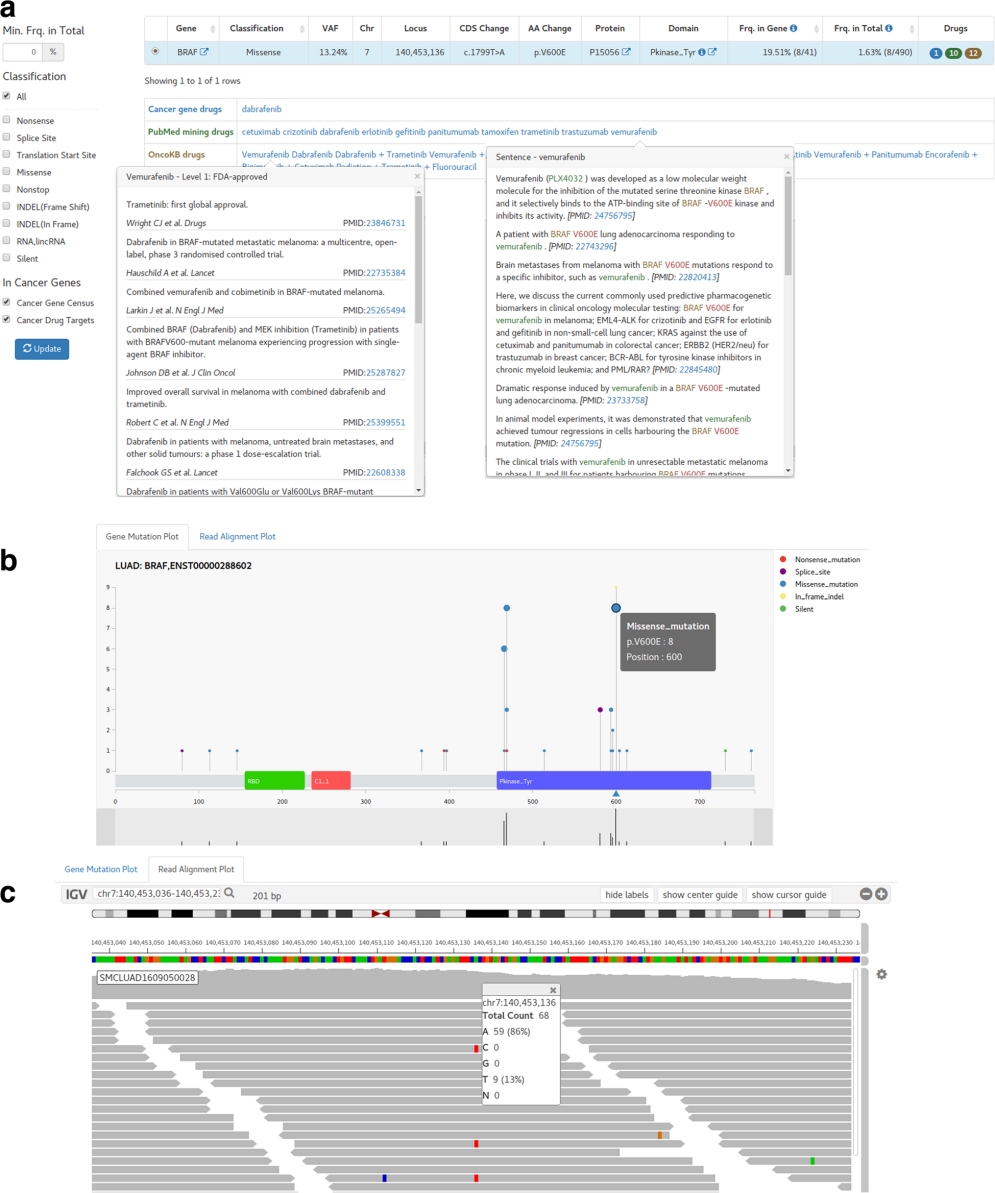
Screen shots of variant reports and exploration (BRAF p.V600E) (**a**). Variant annotation and prioritizing with available drugs. The variant report includes mutation position, variant allele frequency (VAF), patient frequencies, and drugs. Drug table shows mutation-relevant drugs recommended from various resources such as authentic drugs in clinical usage, OncoKB drugs classified in 4 levels, and drugs reported in PubMed abstracts. Filters to show variants of specified properties only are located at left-side. **b** Needle plot of annotated mutations in BRAF gene. Both height and circle size represent the frequency of mutation at each location among the TCGA patient cohort (LUAD in this example). The location and type of mutations found in the patient of the report is indicated by up-triangle icons under the protein sequence bar that contains the protein domains. The bottom part is for zooming in specific area. **c** Reads alignment plot around the mutation point (±100 bp range) is shown in the web browser using IGV java script version

The variant calling process using the WES Galaxy pipeline produces VCF (variant calling format containing details of variants) and BAM (binary alignment map for aligned reads) files, which are imported to the variant annotation and prioritization module of CGIS. We used Oncotator as the main tool for annotating genomic point mutations and short indels [14]. Since many transcripts can be made from the same gene, transcript selection is an important issue in variant annotation. For example, EGFR chr7:55259515 T > G mutation can be annotated as p.L858R only through proper choice of transcript among many different EGFR transcripts. In an effort to resolve this issue, we use the UniProt’s canonical sequence as the reference to collect all transcripts that produce the canonical protein sequence in translation. We further added transcripts concordant with all clinically actionable variants in MyCancerGenome [12]. Resulting list of transcripts was provided to Oncotator [14] with the command line option of (−c) to make these transcripts as primary annotation targets. An example of variant annotation results is shown in Fig. 2a.

Drugs targeting specific variants of the patient are of prime interest. As listed in Table 1, we compiled various resources on cancer drugs for targeted therapies both in clinical usage and in preclinical development. Specifically, we categorized drugs into three groups – 1) in-house curated drugs for actionable targets which include the FDA-approved drugs, 2) drugs reported in PubMed abstracts obtained from systematic text mining and manual curation, and 3) OncoKB [11] drugs that classified drugs in four levels of reliability according to clinical applicability. We carefully characterized (potential) clinically relevant alterations and assigned available drugs to somatic mutations at the variant and gene levels. For the in-house curated drugs for actionable targets, we included the FDA-approved drugs, drugs in clinical trials referenced by highly reliable sources such as MyCancerGenome [12], IntOGen [15], Handbook of targeted cancer therapy [16], and manual searches in the New England Journal of Medicine journal. Drugs from text mining were obtained from VarDrugPub [17] that identified the variant-gene-drug relations in all the PubMed abstracts using a machine learning method.

We further provide filtering utility to select genes of known importance in cancer as well as variants based on patient frequency and functional impact (Fig. 2a). The list of known cancer genes was obtained from the Cancer Gene Census of COSMIC (616 genes) [10]. Users may also select the cancer drug targets in clinical practice (26 genes) that were curated by MD Anderson personalized cancer medicine Knowledgebase [13]. These two sets of cancer genes may be the prime targets of personalized treatment and can be focused by the checkbox filtering as shown in Fig. 2a.

It is often the case that users want to examine the details of specific mutation. We provide three interactive plots for efficient variant exploration. The mutation distribution plot (Fig. 2b) shows the mutation spot on the gene structure with functional domains. Mutation frequency among TCGA patients with the same cancer type is shown in the needle plot format. We also show the read alignment plot (Fig. 2c) so that users can check the validity of mutation calls and allele frequencies. To implement this feature without carrying the large-sized BAM file, our NGS pipeline creates a reduced BAM file that contained the read alignments near the mutation points only. Lastly, we support the co-mutation plot to examine the landscape of somatic mutations and CNVs (Additional file 7: Figure S3). Mutations in a specific patient can be readily compared with the cohort population such as the TCGA data.

In sum, our variant annotation and prioritization scheme based on knowledge of cancer genes and targeted drugs provides an efficient way of scrutinizing clinical relevance of somatic variants in a given cancer type.

### Patient stratification and survival analysis

Proper stratification of patients is the most fundamental concept of targeted precision medicine. We implemented two most commonly used methods of grouping patients based on mutation and gene expression data. Survival analysis of resulting patient groups can be carried out interactively to facilitate hypothesis test of survival benefit for clinicians.

### Mutual exclusivity among driver mutations based on signaling networks

**Fig. 3:**
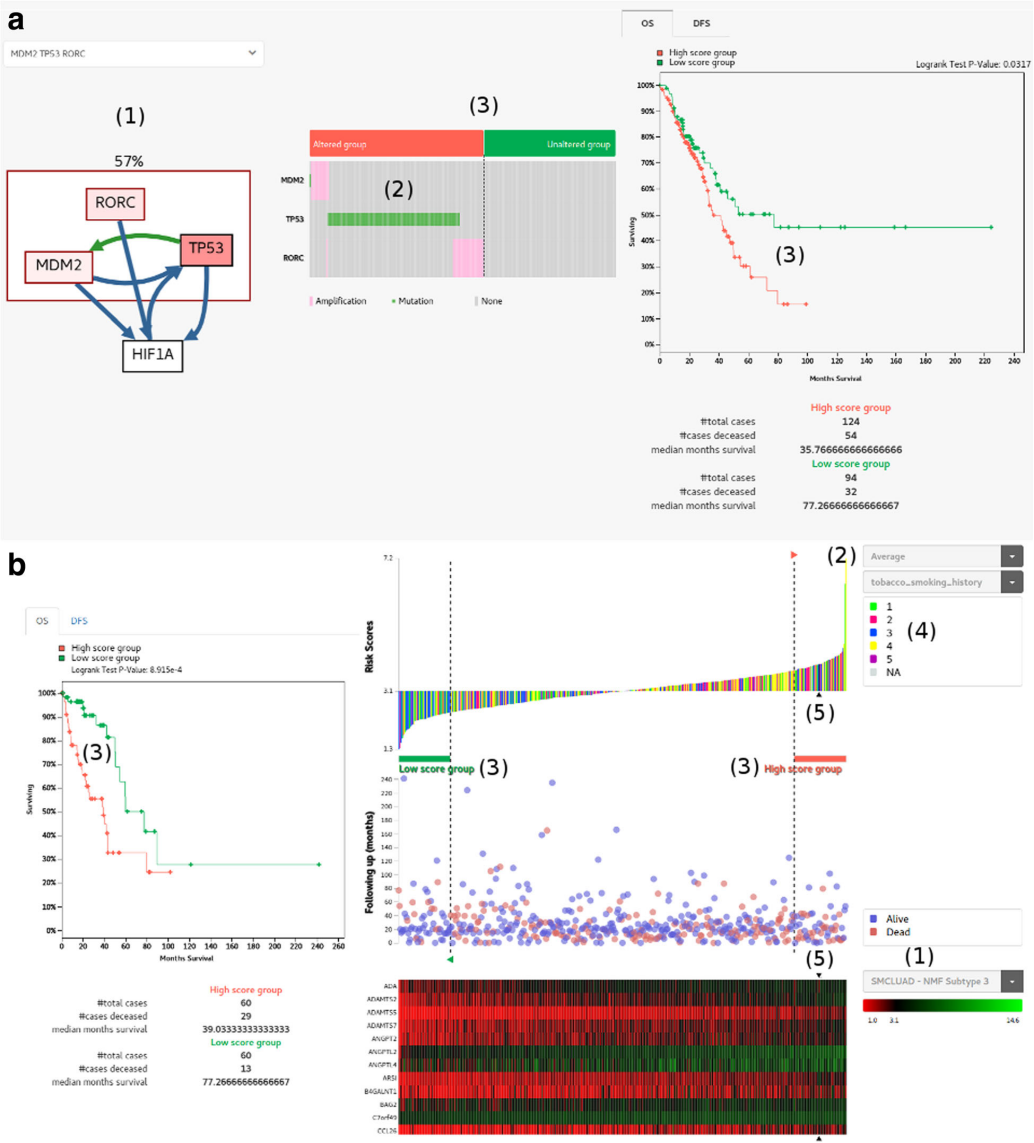
Patient stratification and survival analysis. **a** Patient grouping by mutual exclusivity of a group of gene alterations. (1) As an example, we show a group of mutually exclusively altered genes (RORC, MDM2 and TP53) having a common downstream target (HIF1A) identified by Mutex [18]. Color intensity of each gene is proportional to the alteration ratio. Green and blue edges represent transcriptional relations and post-translational relations, respectively. The patient frequency of alteration in exclusive genes is indicated above the box (i.e. 57%). (2) Distribution of mutations and copy number changes shows the mutually exclusive pattern. (3) Division of patients into two groups of altered and unaltered, and the survival plot between two groups. **b** Patient grouping by gene expression signature. (1) Select the expression signature genes pre-defined for each cancer type (e.g. PAM50 for breast cancer [21]). (2) Decide the mathematical function to calculate the risk score from expression values of signature genes. Samples are sorted according to the risk score as shown in the waterfall plot. (3) Select the high risk and low risk groups by moving dotted vertical lines. The survival plot shows the difference of survival rates between two groups. (4) Clinical or molecular features of patients are mapped onto the waterfall plot. (5) Expression profile of signature genes in the TCGA patient cohort (LUAD in this example). The position of our patient under study is indicated with arrow icons

In tumor, not one but several alternative driver alterations in different genes can lead to similar downstream events. A key observation is that when a member of a substitutive gene set is altered, the selection pressure on the other members is diminished or even nullified. As a result, the mutation pattern of alternative driver genes appears almost mutually exclusive among different patients. We use Mutex program [18] to identify mutually exclusive set of genes with a common downstream effect on the signaling network and implemented survival analysis for altered vs. unaltered patient groups. An example of the TP53 signaling module targeting HIF1A gene is shown in Fig. 3a, taking TCGA LUAD as the patient cohort. Note that the gene alteration includes both somatic mutations and CNVs here.

### Patient grouping by gene expression signatures

DNA sequencing will not be sufficient to optimally select patients for all classes of targeted therapy. In fact, other types of high-throughput technologies, including RNA sequencing, DNA methylation profiling, and small RNA profiling, are being extensively used to identify cancer subtypes and to further improve our understanding of their biological mechanisms. RNA sequencing is the closest to the clinical applications [19]. For example, OncotypeDX based on expression profile of 21 genes predicts accurately recurrence of early-stage ER-positive breast cancer, demonstrating the possibility of molecular prognosis [20]. We implemented a scheme to sort out patients according to the risk score based on expression value of pre-defined genes (Fig. 3b). The score was derived from the average expression value of 103 genes that defined the metastatic subgroup in our in-house study. Patients in the TCGA LUAD cohort were ranked by the score in the waterfall plot, and we defined the highest and lowest 60 patients as high and low score groups respectively. The difference in the overall survival rate between two groups indicates that the corresponding signature genes may have prognostic value in lung adenocarcinoma. Notably, the list of scoring genes and threshold for defining patient groups are provided by users interactively. Thus the system is flexible enough test diverse clinical hypotheses.

### Altered key pathways

**Fig. 4:**
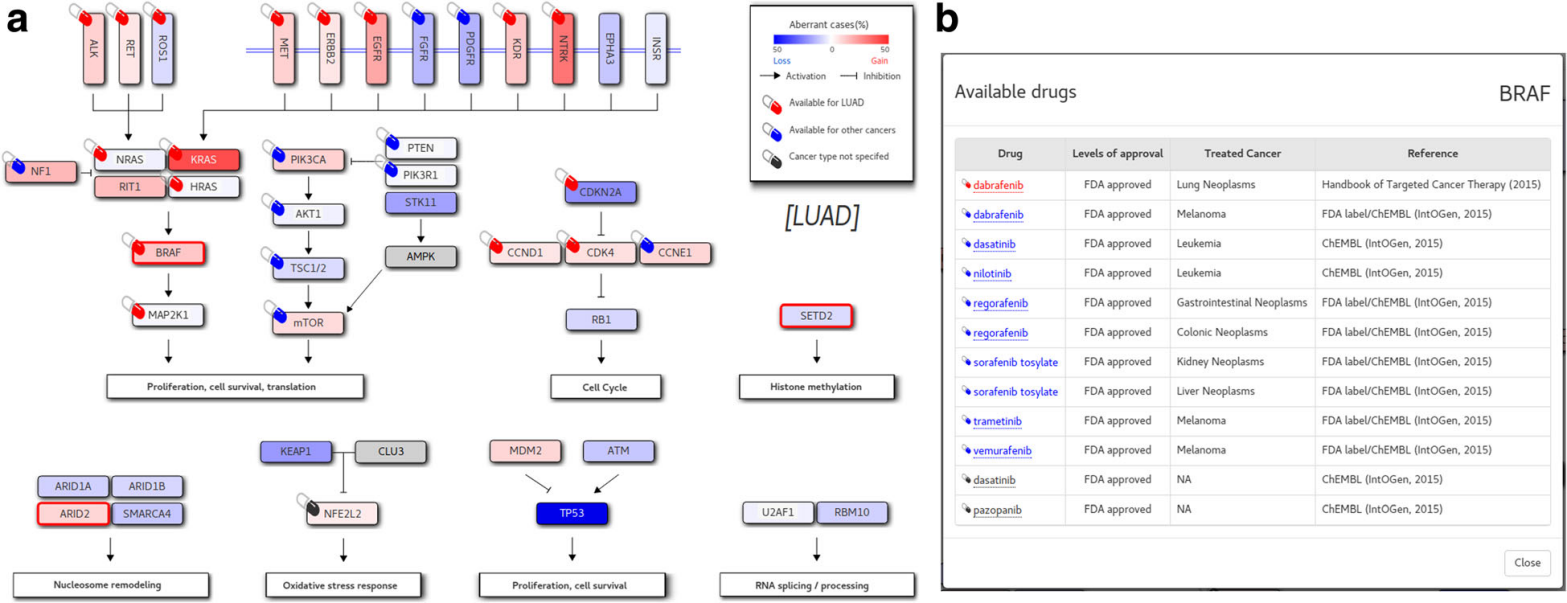
Aberrant key pathways for LUAD. **a** Mutated genes (BRAF, SETD2 and ARD2 in this case) in the given patient are indicated in thick red border. The background color is determined by the CNAs (gain in read and loss in blue), with the color depth reflecting the frequency of patients who were affected by mutation or CNAs. **b** A click on a pill icon opens up a window that shows available drugs targeting the gene of interest (BRAF in this example). Drugs are color-coded according to the approval status

The eventual development of acquired resistance has been a near universal observation with targeted cancer therapy. Even in patient samples where those acquired resistance emerges, alterations often converge on specific gene modules or pathways, suggesting that even these scenarios could be managed with drugs or drug combinations that target this biochemical and signaling bottleneck [19]. To address this scenario, we defined and unified the altered key pathways for each cancer type that demonstrate how multiple signaling pathways interact via cross-talk and feedback. An example of altered key pathways is shown in Fig. 4a for lung adenocarcinoma. Note that genes are colored according to the abundance of activating or suppressing aberrations (mutations and CNAs). Drugs targeting each gene in the pathway are also listed to help users search available drugs targeting genes on up- or down-stream path (Fig. 4b).

## Conclusion

Our CGIS software was designed both for clinicians seeking for an easy-to-understand report of genomic analysis and for medical scientists who want to explore genomic information to test clinical hypotheses for biomarker development. We integrated ample genomic information from diverse public resources with manual curation if necessary. We also devised and implemented several novel ideas and tools for investigating roles of variants, exploring population cohorts, patient stratification based on genomic data, and drugs based on pathway view. This is just a prototype result of our project and we will continue to develop more features and modules for enhanced function and convenience.

### Availability and requirements

Project name: Clinical and Genomic Information System (CGIS) for cancer precision medicine.

Project home page: http://203.255.191.21

Operating system(s): Platform independent.

Programming language: JavaScript.

Other requirements: Node.js version 4.4.7 or higher, MySQL version 5.6 or higher, D3.js, igv.js.

License: Proprietary (allowed for non-commercial use only).

Any restrictions to use by non-academics: restricted by the license.

## Additional files

Additional file 1: **Figure S1.** Galaxy workflow for WES data processing. (PNG 221 kb)

Additional file 2: **Figure S2.** Galaxy workflow for WTS data processing. (PNG 111 kb)

Additional file 3: Galaxy workflow file (json data format) for WES data processing, it can be imported to another Galaxy server. (GA 53 kb)

Additional file 4: Galaxy workflow file (json data format) for WTS data processing, it can be imported to another Galaxy server. (GA 23 kb)

Additional file 5: Instruction for users to upload their own FASTQ files into our BioCloud system so that they can process the NGS data and get the various reports described in main script. (PDF 1060 kb)

Additional file 6: **Figure S4.** An example of filtering process to select a patient cohort based on clinical information or properties. A. Selection of female and lifelong never-smoker patients in the TCGA LUAD cohort. (“Cohort Selection” menu is located in left-top side of the page) B. Driver genes were sorted by mutation frequency by clicking the “# Mutations” label at the bottom. The sorting result confirmed that EGFR is the most frequently mutated gene among these patients, whereas TP53 mutation was prevalent in other patients as shown in Additional file 7: Figure S3. (PNG 179 kb)

Additional file 7: **Figure S3.** Cohort explorer for the whole TCGA LUAD cohort and our patient (1) Significant driver genes identified by MutSigCV [22]. Each horizontal bar represents total count of mutations on the corresponding gene in the cohort. Color scheme indicates the coding properties of mutations. (2) The gray bar represents –log_10_(p-values) of each driver gene. (3) Sample-wise count of mutations with coding properties color-coded. (4) Clinical features of samples. (5) Mutations found in our patient are plotted at left-most side (i.e. the first column). (PNG 120 kb)

## Abbreviations

BAM: Binary Alignment Map
CNA: Copy Number Aberration
CNV: Copy Number Variation
INDEL: Insertion or Deletion
LUAD: Lung Adenocarcinoma
NGS: Next Generation Sequencing
SNV: Sing Nucleotide Variant,
TCGA: The Cancer Genome Atlas,
VCF: Variant Call Format
WES: Whole Exome Sequencing
WTS: Whole Transcriptome Sequencing
