# Supplementary figures and images for: An integrated clinical and genomic information system for cancer precision medicine
Source: BMC Med Genomics. 2018 Apr 20;11(Suppl 2):34. doi: 10.1186/s12920-018-0347-9 (PMC5918454; doi:10.1186/s12920-018-0347-9)

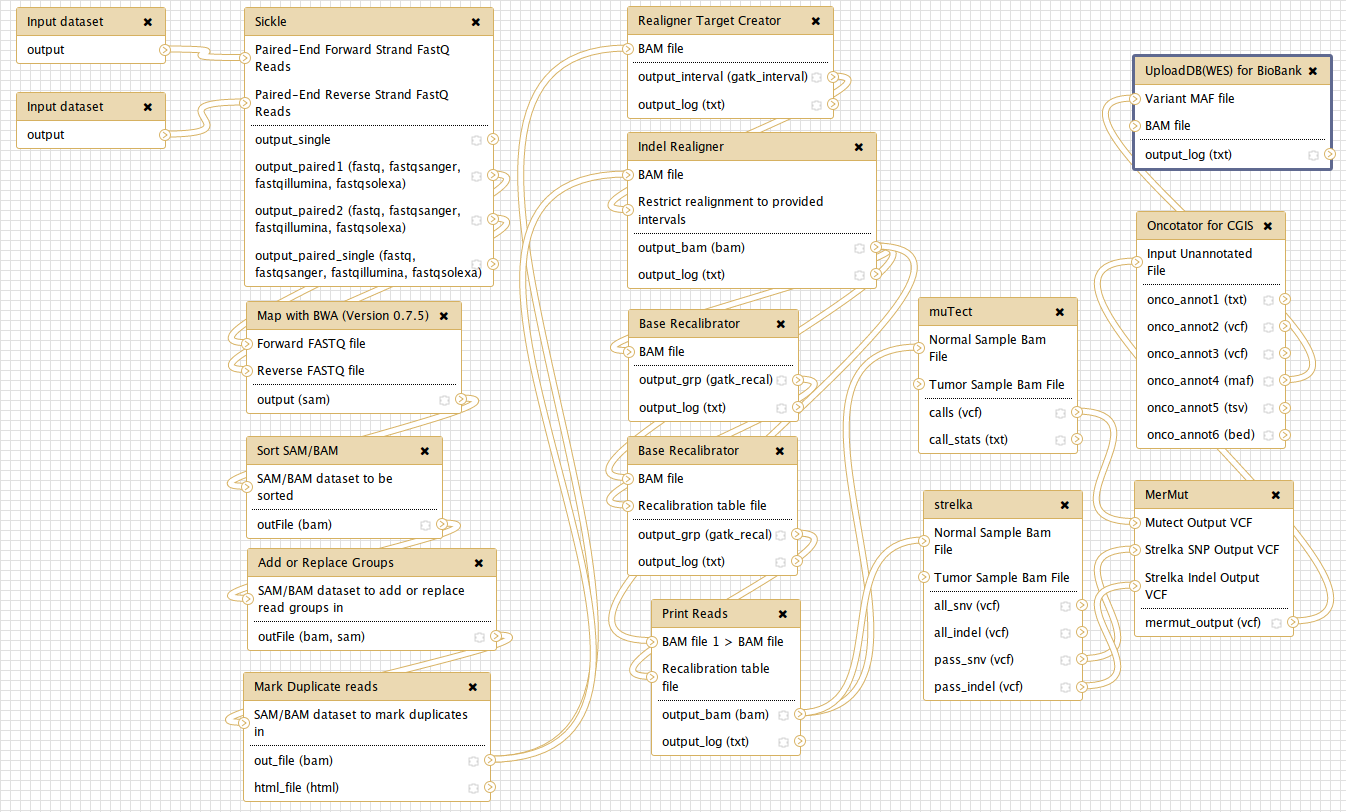

Supplement: Supplementary file 1 — Figure S1. Galaxy workflow for WES data processing. (PNG 221 kb) [file 12920_2018_347_MOESM1_ESM.png]

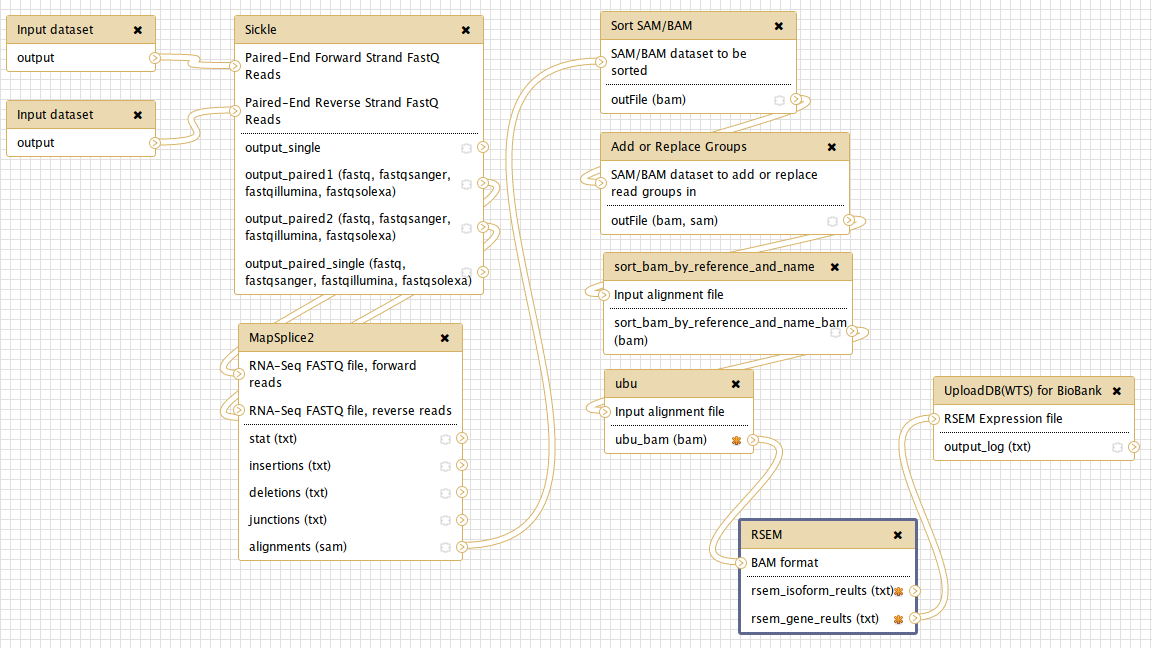

Supplement: Supplementary file 2 — Figure S2. Galaxy workflow for WTS data processing. (PNG 111 kb) [file 12920_2018_347_MOESM2_ESM.png]

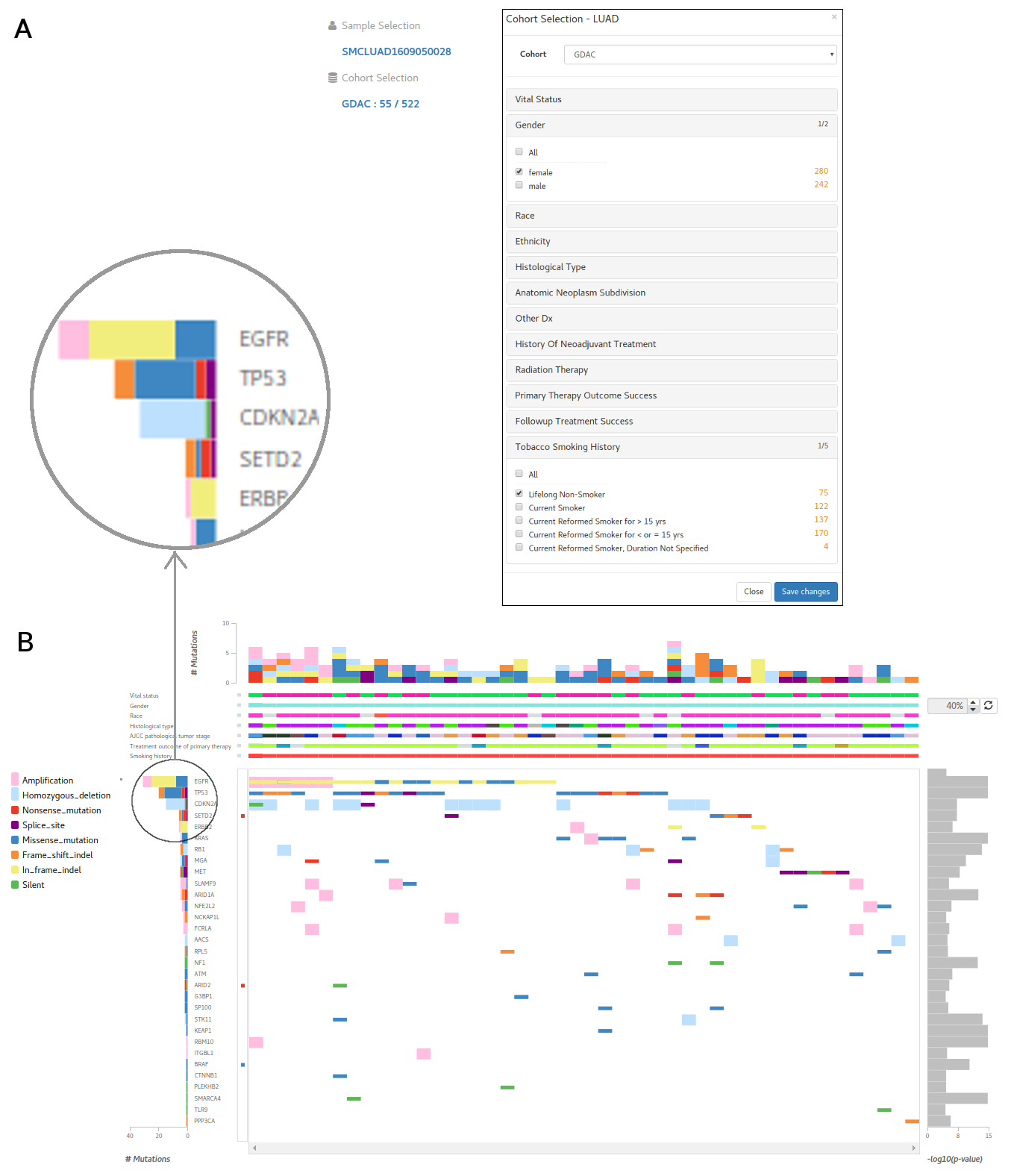

Supplement: Supplementary file 6 — Figure S4. An example of filtering process to select a patient cohort based on clinical information or properties. A. Selection of female and lifelong never-smoker patients in the TCGA LUAD cohort. (“Cohort Selection” menu is located in left-top side of the page) B. Driver genes were sorted by mutation frequency by clicking the “# Mutations” label at the bottom. The sorting result confirmed that EGFR is the most frequently mutated gene among these patients, whereas TP53 mutation was prevalent in other patients as shown in Additional file 7: Figure S3. (PNG 179 kb) [file 12920_2018_347_MOESM6_ESM.png]

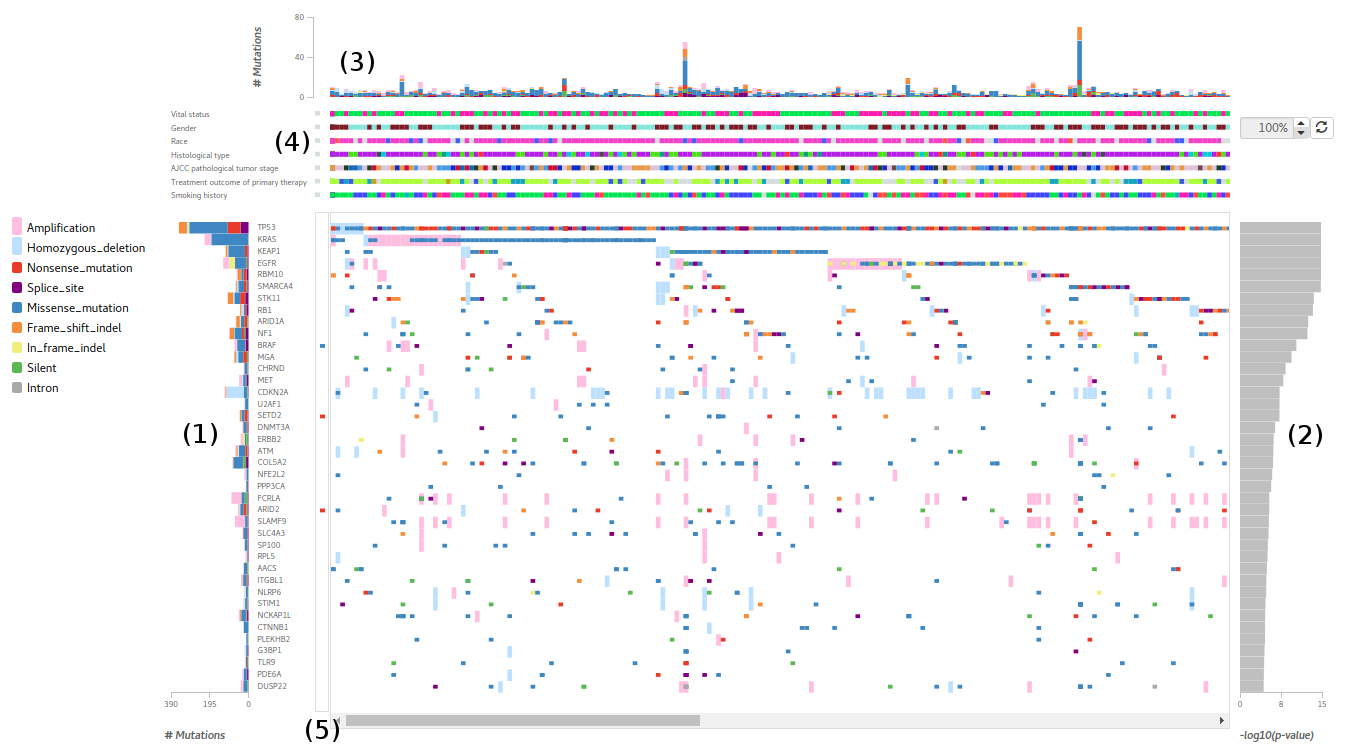

Supplement: Supplementary file 7 — Figure S3. Cohort explorer for the whole TCGA LUAD cohort and our patient (1) Significant driver genes identified by MutSigCV [22]. Each horizontal bar represents total count of mutations on the corresponding gene in the cohort. Color scheme indicates the coding properties of mutations. (2) The gray bar represents –log10(p-values) of each driver gene. (3) Sample-wise count of mutations with coding properties color-coded. (4) Clinical features of samples. (5) Mutations found in our patient are plotted at left-most side (i.e. the first column). (PNG 120 kb) [file 12920_2018_347_MOESM7_ESM.png]
