# Supplementary material for: An integrated clinical and genomic information system for cancer precision medicine
Source: BMC Med Genomics. 2018 Apr 20;11(Suppl 2):34. doi: 10.1186/s12920-018-0347-9 (PMC5918454; doi:10.1186/s12920-018-0347-9)
Supplement: Supplementary file 5 — Instruction for users to upload their own FASTQ files into our BioCloud system so that they can process the NGS data and get the various reports described in main script. (PDF 1060 kb) [file 12920_2018_347_MOESM5_ESM.pdf]

Information System for Cancer Precision Medicine

Oct 13, 2017
Yeongjun Jang <gentie@snu.ac.kr>

이화여자대학교
EWHA WOMANS UNIVERSITY

ERCS 이화시스템생물학연구소
Ewha Research Center for Systems Biology

Log-in to the BioCloud storage

- Download WinSCP at here
- Log-in to our BioCloud system
  - Server IP address: **203.255.191.225**
  - ID: **SMC**
  - Password: **SMC101**
- Connection preferences
  - File protocol: **FTP**
  - Encryption: **TLS/SSL Explicit encryption**

Log-in to the BioCloud storage

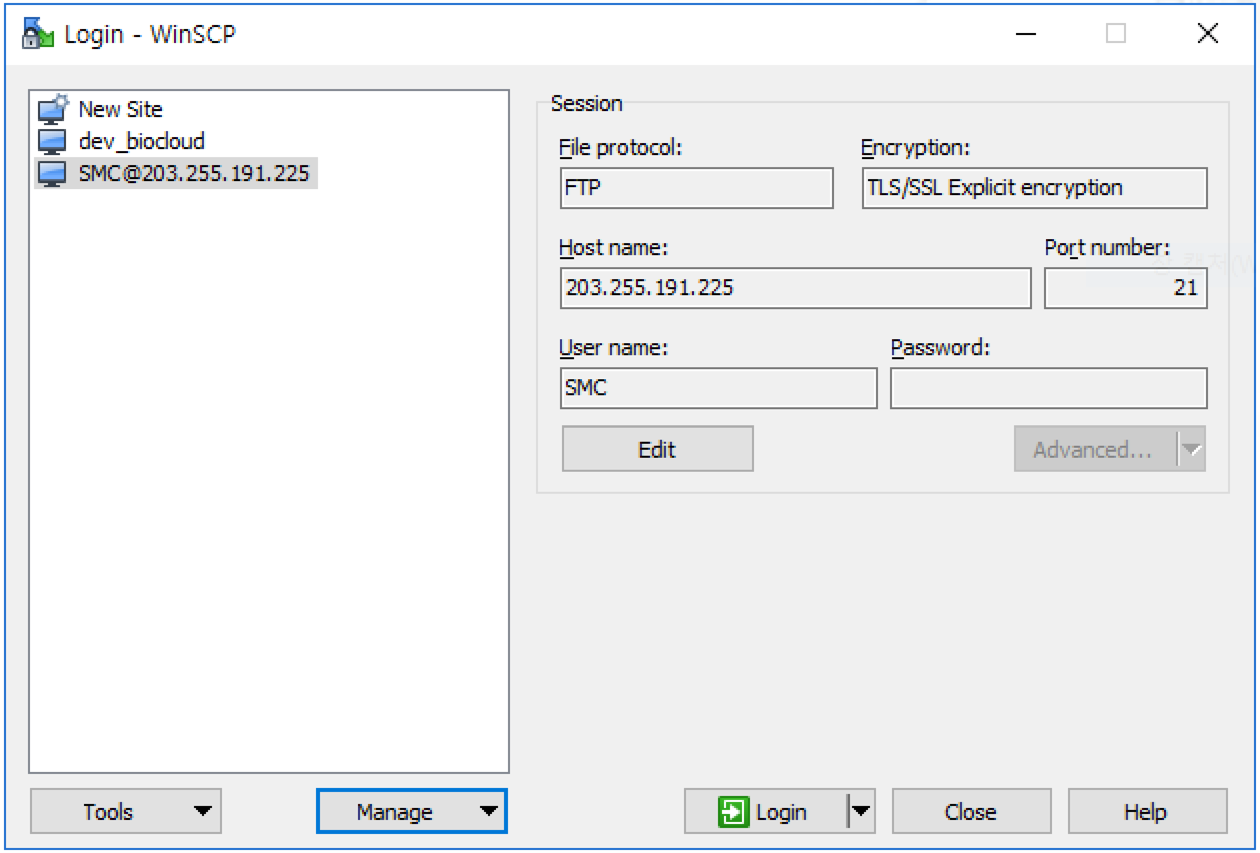

Upload FASTQ files

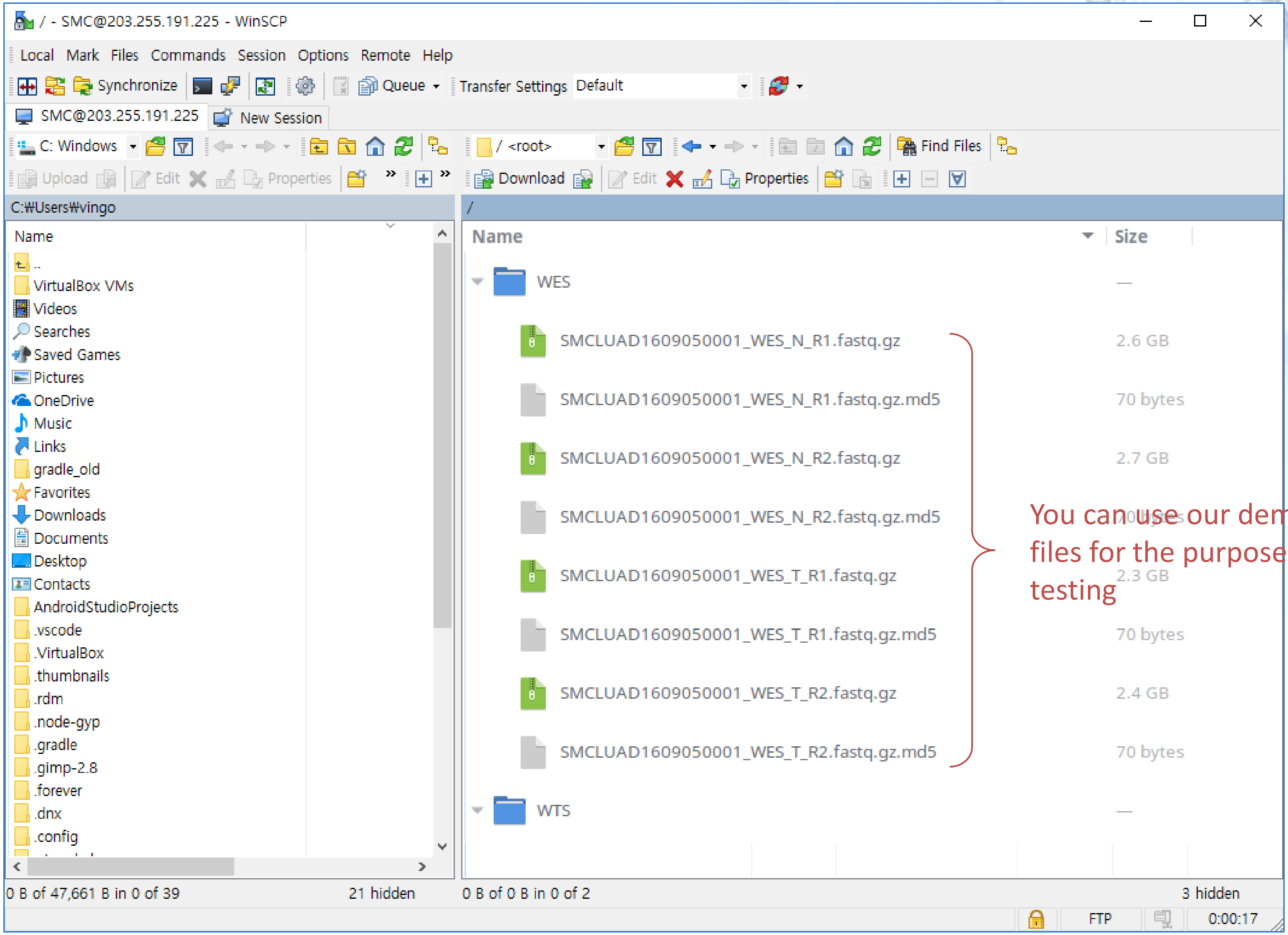

You can use our demo files for the purpose of testing

Log-in to CGIS

http://203.255.191.21

ID: demo@gmail.com

Password: showme

Log-in to CGIS

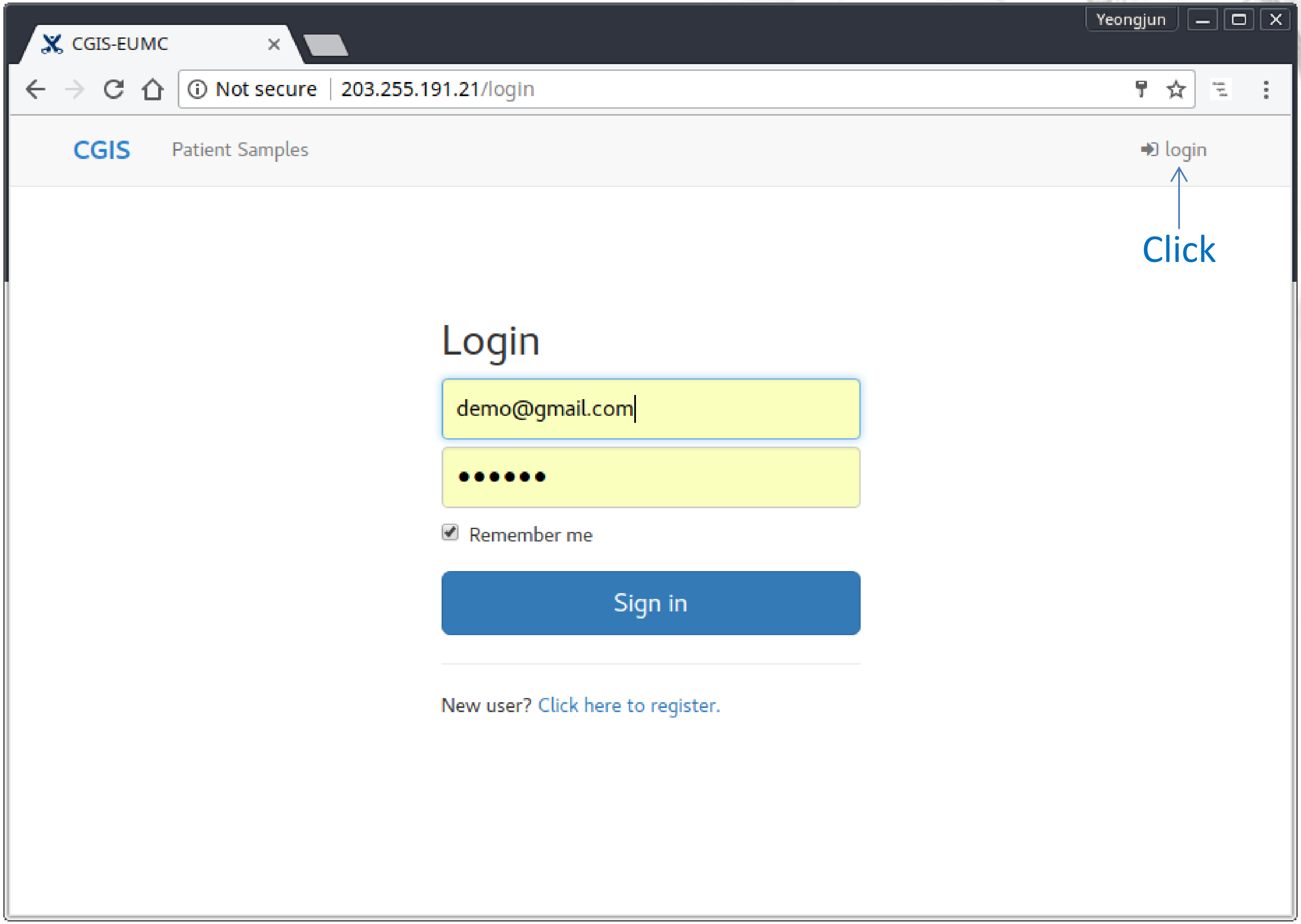

Page of sample list

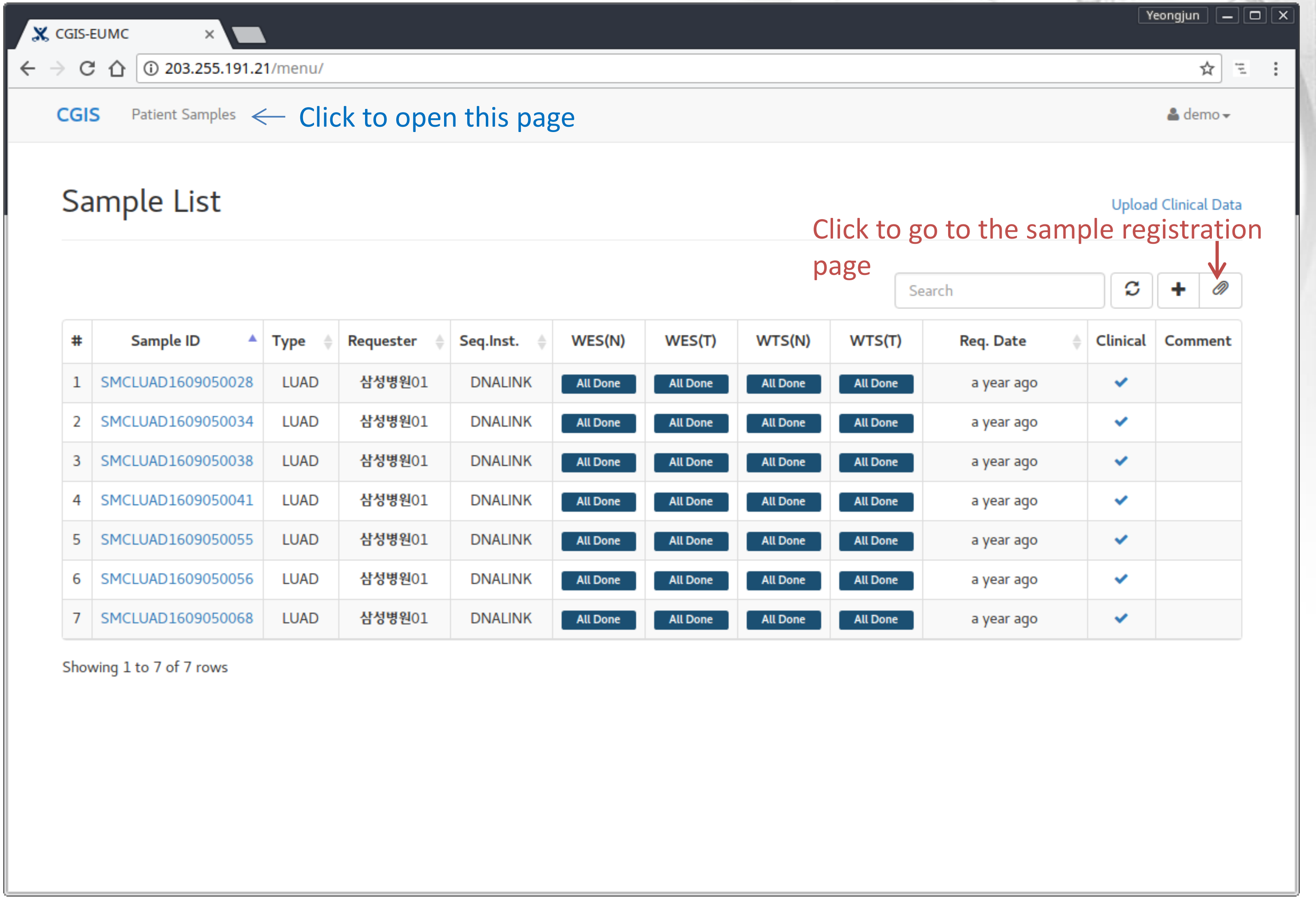
CGIS-EUMC
203.255.191.21/menu/
Yeongjun
CGIS Patient Samples ← Click to open this page
demo

Sample List

Upload Clinical Data

Click to go to the sample registration page

Search

#	Sample ID	Type	Requester	Seq.Inst.	WES(N)	WES(T)	WTS(N)	WTS(T)	Req. Date	Clinical	Comment
1	SMCLUAD1609050028	LUAD	삼성병원01	DNALINK	All Done	All Done	All Done	All Done	a year ago	✔	
2	SMCLUAD1609050034	LUAD	삼성병원01	DNALINK	All Done	All Done	All Done	All Done	a year ago	✔	
3	SMCLUAD1609050038	LUAD	삼성병원01	DNALINK	All Done	All Done	All Done	All Done	a year ago	✔	
4	SMCLUAD1609050041	LUAD	삼성병원01	DNALINK	All Done	All Done	All Done	All Done	a year ago	✔	
5	SMCLUAD1609050055	LUAD	삼성병원01	DNALINK	All Done	All Done	All Done	All Done	a year ago	✔	
6	SMCLUAD1609050056	LUAD	삼성병원01	DNALINK	All Done	All Done	All Done	All Done	a year ago	✔	
7	SMCLUAD1609050068	LUAD	삼성병원01	DNALINK	All Done	All Done	All Done	All Done	a year ago	✔	

Showing 1 to 7 of 7 rows

Sample Registration

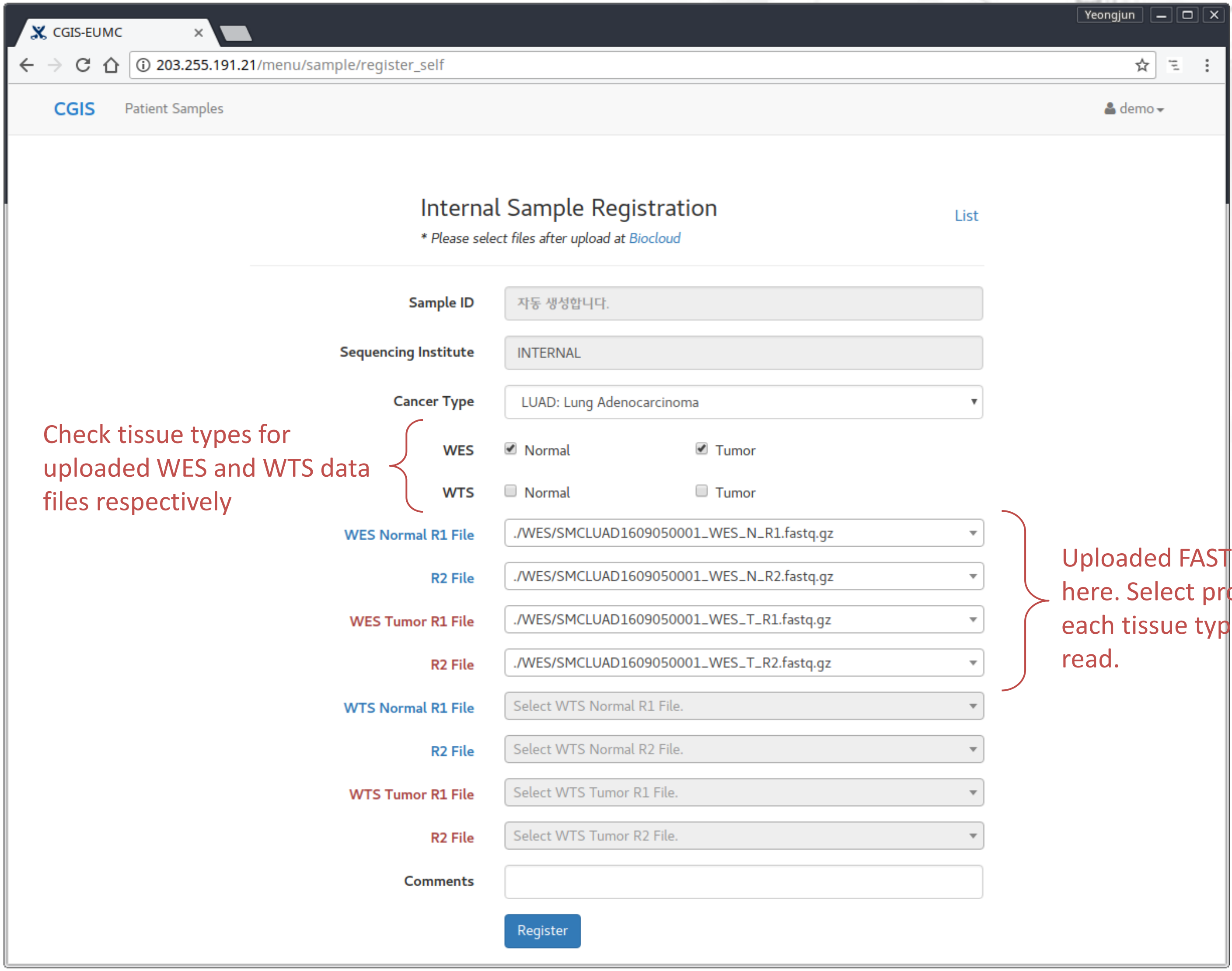

Check tissue types for uploaded WES and WTS data files respectively

Uploaded FASTQ files are listed here. Select proper FASTQ files for each tissue type and paired-end read.

Sample Registration (Result)

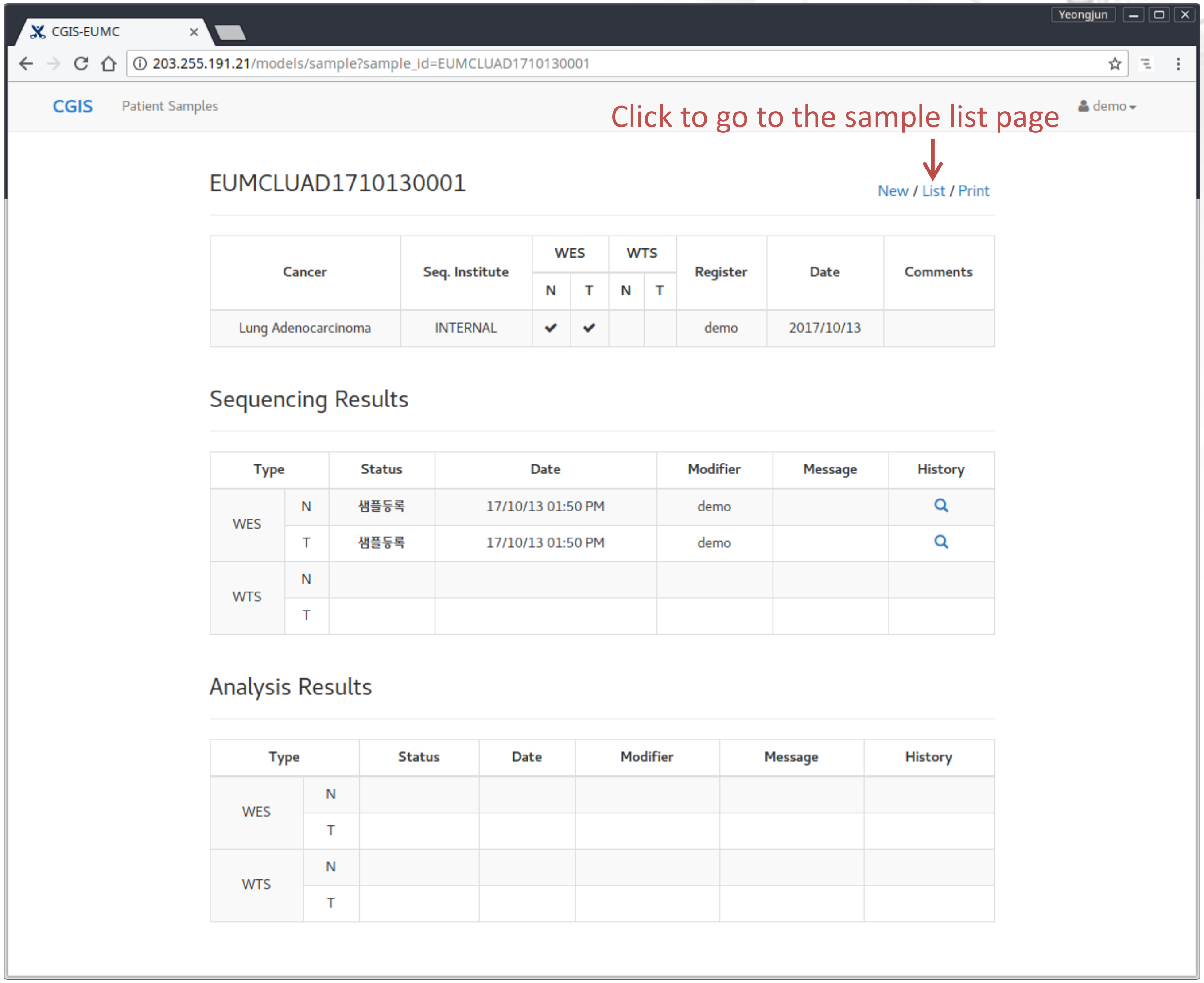

CGIS-EUMC

203.255.191.21/models/sample?sample_id=EUMCLUAD1710130001

CGIS Patient Samples demo

Click to go to the sample list page

EUMCLUAD1710130001

New / List / Print

Cancer	Seq. Institute	WES		WTS		Register	Date	Comments
		N	T	N	T			
Lung Adenocarcinoma	INTERNAL	✔	✔			demo	2017/10/13	

Sequencing Results

Type		Status	Date	Modifier	Message	History
WES	N	샘플등록	17/10/13 01:50 PM	demo		🔍
	T	샘플등록	17/10/13 01:50 PM	demo		🔍
WTS	N					
	T					

Analysis Results

Type		Status	Date	Modifier	Message	History
WES	N					
	T					
WTS	N					
	T					

Sample Registration (Result)

** After the pipeline is completed, you should upload a file containing clinical information for the sample. This is a mandatory step for getting the report of results

Click to go to the page for monitoring the analysis process

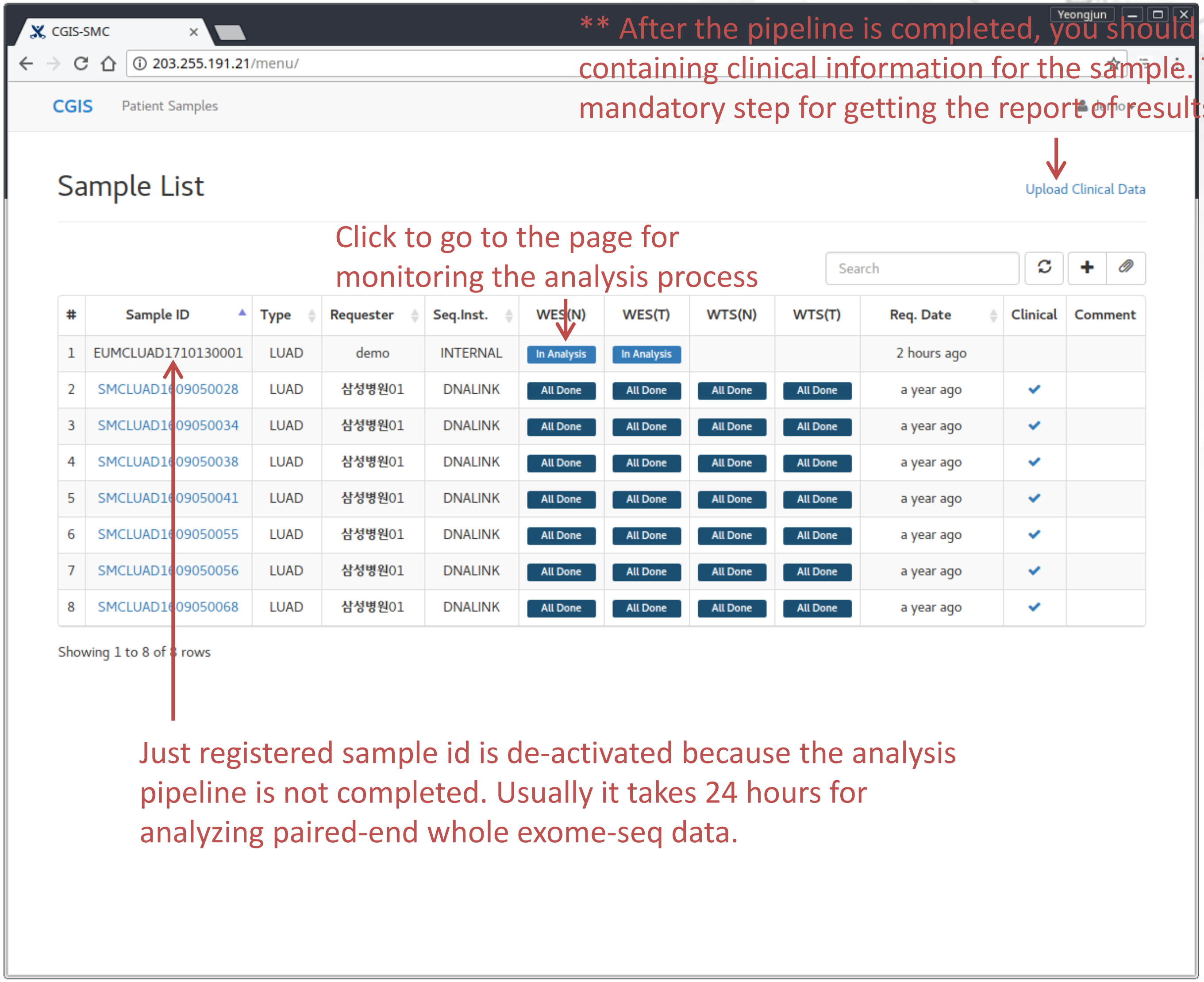

#	Sample ID	Type	Requester	Seq.Inst.	WES(N)	WES(T)	WTS(N)	WTS(T)	Req. Date	Clinical	Comment
1	EUMCLUAD1710130001	LUAD	demo	INTERNAL	In Analysis	In Analysis			2 hours ago		
2	SMCLUAD1609050028	LUAD	삼성병원01	DNALINK	All Done	All Done	All Done	All Done	a year ago	✔	
3	SMCLUAD1609050034	LUAD	삼성병원01	DNALINK	All Done	All Done	All Done	All Done	a year ago	✔	
4	SMCLUAD1609050038	LUAD	삼성병원01	DNALINK	All Done	All Done	All Done	All Done	a year ago	✔	
5	SMCLUAD1609050041	LUAD	삼성병원01	DNALINK	All Done	All Done	All Done	All Done	a year ago	✔	
6	SMCLUAD1609050055	LUAD	삼성병원01	DNALINK	All Done	All Done	All Done	All Done	a year ago	✔	
7	SMCLUAD1609050056	LUAD	삼성병원01	DNALINK	All Done	All Done	All Done	All Done	a year ago	✔	
8	SMCLUAD1609050068	LUAD	삼성병원01	DNALINK	All Done	All Done	All Done	All Done	a year ago	✔	

Just registered sample id is de-activated because the analysis pipeline is not completed. Usually it takes 24 hours for analyzing paired-end whole exome-seq data.

Analysis Monitoring

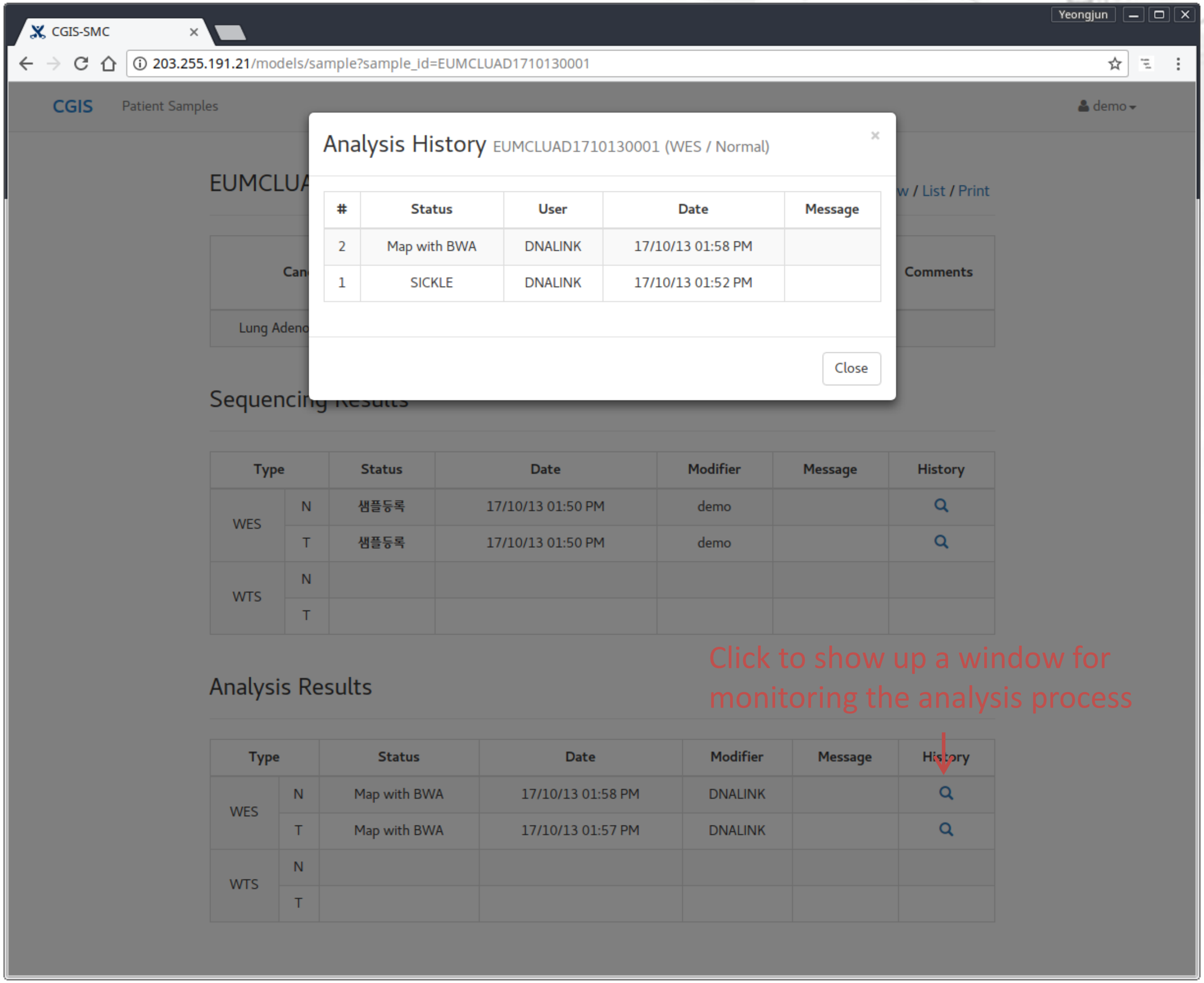

Upload Clinical Data

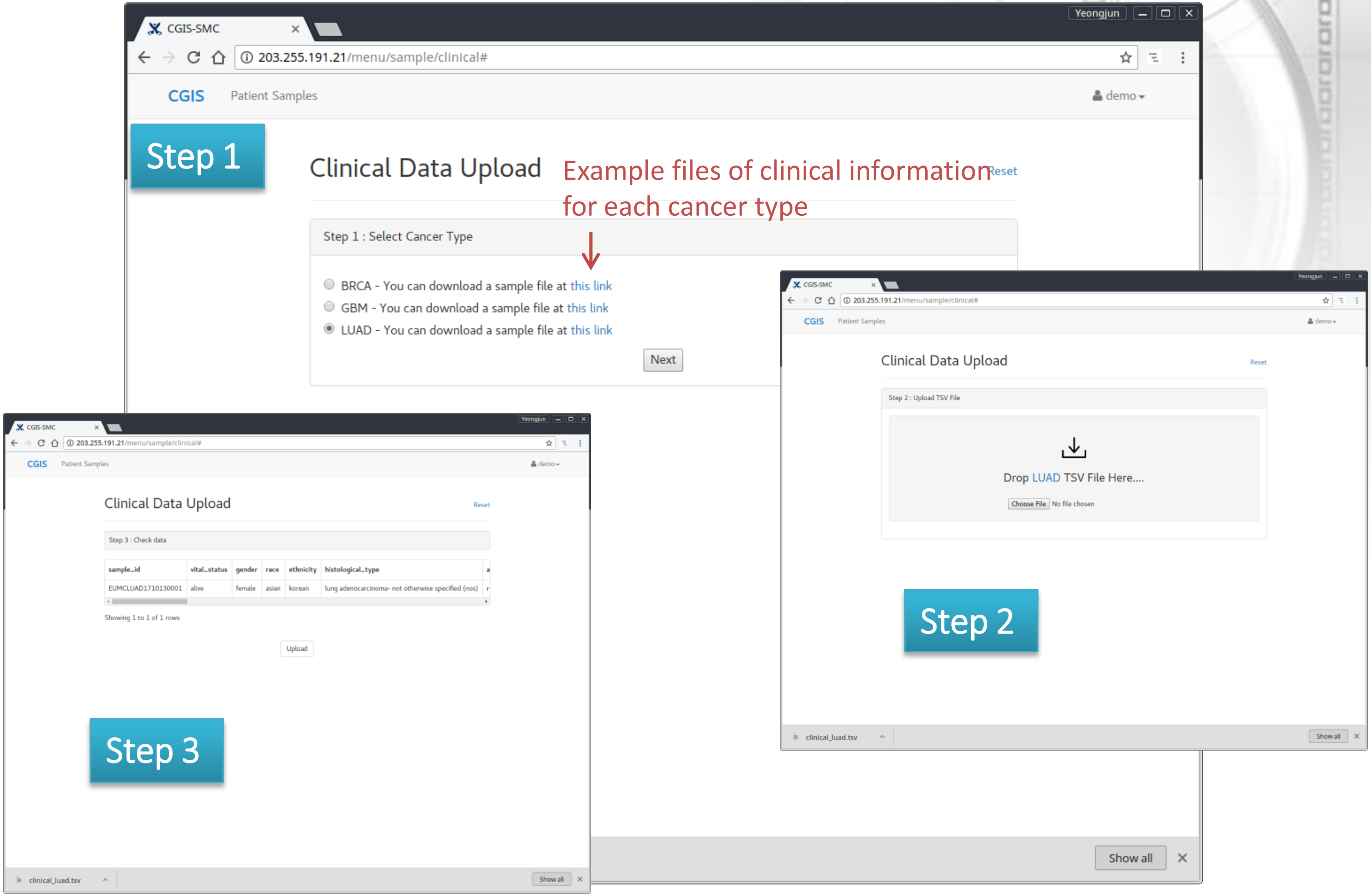

END

gentie@snu.ac.kr
